# A Case of Diabetic Ketoacidosis Complicated With Necrotizing Esophagitis

**DOI:** 10.7759/cureus.52871

**Published:** 2024-01-24

**Authors:** Shumei Kawashima, Hironori Abe, Norihiro Shimizu, Junpei Shikuma, Ryo Suzuki

**Affiliations:** 1 Diabetes and Endocrinology, Tokyo Medical University, Tokyo, JPN

**Keywords:** hematemesis, black esophagus, diabetic keto acidosis, type 1 diabetes mellitus (t1d), acute necrotizing esophagitis

## Abstract

Acute necrotizing esophagitis (ANE) is known as the "black esophagus." We present a case of ANE in a patient with slowly progressive type 1 diabetes mellitus. A 49-year-old man presented with vomiting, characterized by coffee residue-like emesis, and was diagnosed with diabetic ketoacidosis. Upper gastrointestinal endoscopy revealed black mucosa extending from the middle of the esophagus to the gastric junction, leading to a diagnosis of ANE. The patient was treated with proton pump inhibitors and showed marked improvement. The patient was discharged on the 20th day of illness.

## Introduction

Acute necrotizing esophagitis (ANE), also known as the "black esophagus," was first described by Goldenberg et al. in 1990 following cholecystectomy [1]. It is a rare disease, with only 30 cases reported in Japan until 2014. Its etiology is unknown, although ischemia is believed to be involved [2]. ANE typically occurs in patients with underlying diseases and is often triggered by serious conditions such as diabetic ketoacidosis (DKA), dehydration, or shock [3]. Treatment of ANE includes fasting, fluid replacement, and the administration of gastric antisecretory agents [4]. The prognosis is generally good if the disease progresses without any complications [5]. In this report, we present a case of ANE in a patient with slowly progressive type 1 diabetes mellitus who developed DKA and presented with black vomiting and a black esophagus on upper gastrointestinal endoscopy.

## Case presentation

A 49-year-old man was diagnosed with slowly progressive type 1 diabetes mellitus at age 35. He had been receiving tonic insulin therapy (48 U), and his HbA1c level was approximately 8.0%. The day before admission (November X-1, 2022), the patient had consumed valuable meat for dinner, and from the next day (X), he experienced nausea and frequent coffee residue-like vomiting. Because his condition did not improve, the patient requested emergency medical assistance on day X+1 and was urgently transported to our hospital. Upon arrival, the patient was conscious and alert. His blood pressure was 126/76 mmHg, pulse 98/min, respiratory rate of 15/min with an O_2_ saturation of 100% on room air. On physical examination, the abdomen was flat, and soft, and no tenderness. But blood tests revealed the following: blood glucose level, 770 mg/dL; total ketone, 16101 μmoL/L; acetoacetate, 11810 μmoL/L; 3-hydroxybutyrate, 4291 μmoL/L; urinary ketone, 3+; and pH, 7.317. Thus, the results indicated marked hyperglycemia and metabolic acidosis, which led to a DKA diagnosis (Table 1).

**Table 1:** Blood test and urinalysis results at the time of hospitalization

Laboratory value	Reference range	Result
White blood cell count (/μL)	2700-8800	17.6×10^3^
Red blood cell count (/μL)	3.7-5.4×10^6^	4.65×10^6^
Hemoglobin (g/dL)	11.0-17.0	15.3
Hematocrit (%)	34.0-49.0	44.3
Platelet count (/μL)	140.0-340.0×10^3^	294×10^3^
Total protein (g/dL)	6.6-8.2	4.5
Albumin (g/dL)	3.9-4.9	4.9
Aspartate aminotransferase (IU/L)	7-38	255
Alanine aminotransferase (IU/L)	4-44	215
Creatine kinase (IU/L)	59-248	52
Blood urea nitrogen (mg/dL)	8.0-22.6	37
Sodium (mEq/L)	138-148	124
Potassium (mEq/L)	3.6-5.2	5.3
Chlorine (mEq/L)	98-108	77
Creatinine (mg/dL)	0.4-0.8	1.1
Glucose (mg/dL)	60-110	770
HbA1c (glycated hemoglobin)(%)	4.6-6.2	7.3
Glycoalbumin (%)	11.8-16.3	23
Amylase (U/L)	39-124	38
Total ketones (μmol/L)	26-122	16101
Acetoacetic acid (μmol/L)	13-69	11810
3-hydroxybutyric acid (μmol/L)	0-76	4291
Venous blood gas		
pH	7.33-7.41	7.32
pCO_2 _(mmHg)	43-53	21.6
HCO_3_- (mmol/L)	24-28	10.8
Base excess (mmol/L)	0-4	-12.9
Urinalysis		
Protein	Negative	Negative
Glucose	Negative	3+
Ketone body	Negative	3+

**Figure 1:**
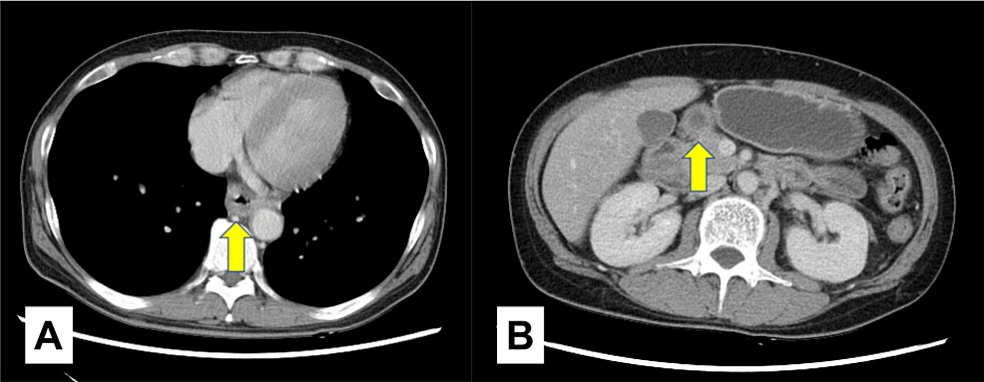
Contrast-enhanced computed tomography of the thorax and abdomen (horizontal section) A: Edematous thickening of the esophageal wall; B: Esophageal and gastric dilatation with pyloric extension

Contrast-enhanced computed tomography of the patient's thorax and abdomen revealed that the esophagus was thickened and edematous, with dilatation of the esophagus and stomach, especially in the pyloric region (Figure 1).

**Figure 2:**
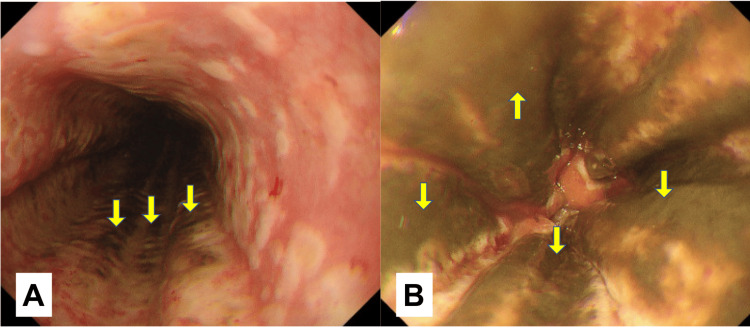
Upper gastrointestinal endoscopy (day zero) A is the lower part of the esophagus. B is the esophagogastric junction. The lower part of the esophagus is covered with dark mucosa and a longitudinal white moss.

Gastrointestinal endoscopy was performed on the same day, and black mucosa was observed from the middle of the esophagus to the junction with the stomach (Figure 2).

**Figure 3:**
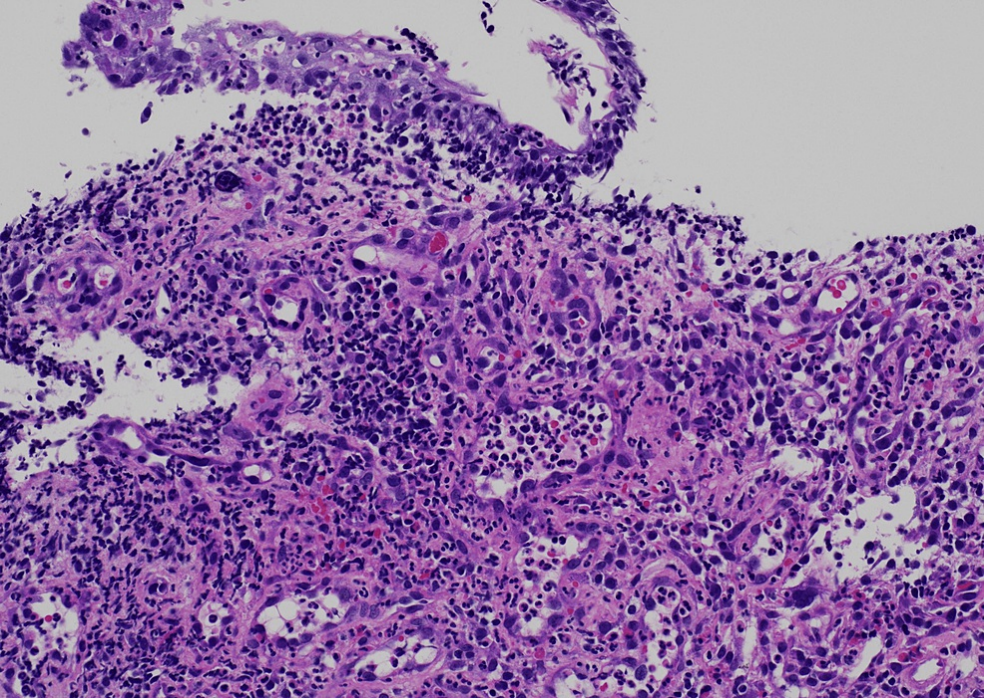
Biopsy of esophageal mucosa (hematoxylin and eosin staining, 100× magnification) Chronic inflammation with acanthotic change is observed. No malignant findings.

Pathological analysis revealed granulation tissue with inflammatory cell infiltration and hypervascularization, leading to an ANE diagnosis (Figure 3).

**Figure 4:**
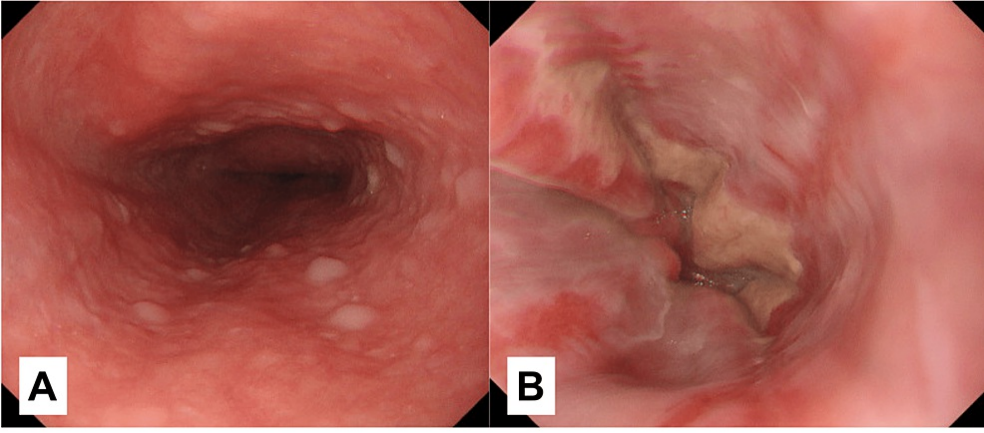
Upper gastrointestinal endoscopy (day nine) A is the lower part of the esophagus. B is the esophagogastric junction. Significant improvement in esophageal mucosa was observed.

The patient was treated for DKA with CVII and adequate fluids, which resolved acidosis the following day. For ANE, the patient was managed with fasting, central venous nutrition, and a proton pump inhibitor (PPI), resulting in significant improvement in the esophageal mucosa on gastrointestinal endoscopy on the ninth day (Figure 4).

On the 13th day, the patient resumed eating and was switched from CVII to intensive insulin therapy, maintaining blood glucose levels between 100 and 200 mg/dL before each meal. The patient showed good progress without any recurrence or complications and was discharged on the 20th day after admission. After being discharged from the hospital, the patient underwent intensive insulin therapy (with a total daily dose of insulin at 52 units), and his HbA1c has consistently remained around 8.0%. To date, there has been no recurrence of ANE, and there have been no complications related to esophageal stricture.

## Discussion

ANE was first reported by Goldenberg et al. in 1990 [1]. ANE etiology remains unknown, but it is suggested that compromised circulation in the lower esophagus, disruption of mucosal defense mechanisms, and exposure to gastric acid may be involved in its development [2]. ANE frequency is estimated to range from 0.013% to 0.28%; in Japan [6-8], there were only 30 reported cases until 2014, indicating its rarity. Two large retrospective series that have reviewed the findings in >100000 endoscopies have estimated the incidence at approximately 0.01% (12 patients) [9,10], and another retrospective analysis of 10295 endoscopies has shown an incidence of 0.28% (29 patients) [4]. This is more commonly observed in females, with a male-to-female ratio of 4:12. The initial symptoms of ANE are hematemesis (blood vomiting), and abdominal pain and nausea may also be present. Many cases of ANE are associated with underlying conditions and often occur because of severe conditions such as DKA, dehydration, or shock [2]. Factors such as the administration of antibiotics, mechanical damage from nasogastric tubes, and gastrointestinal obstruction are also considered to be involved in the development of ANE [11,12]. In the present case, the patient had underlying slowly progressive insulin-dependent diabetes mellitus complicated with DKA. In DKA, gastric dilatation, atony, and rapid hyperglycemia can cause a decrease in lower esophageal pressure and abnormal esophageal peristalsis [3]. Additionally, hyperglycemia can lead to delayed gastric emptying [13], even in the absence of autonomic nervous system disorders, resulting in gastroprokinetic disorders. These factors, combined with increased intraesophageal pressure, reflux of gastric acid into the esophagus, dehydration-induced ischemia of the esophageal mucosa, and microcirculatory disturbances in the lower esophagus caused by DKA, may contribute to ANE development. A search using the keywords "acute necrotizing esophagitis" and "type 1 diabetes mellitus" in PubMed, a medical journal database, yielded eight reports, including autopsy cases (Table 2).

**Table 2:** Cases identified after a search using the keywords "acute necrotizing esophagitis" and "type 1 diabetes mellitus" in PubMed DKA: Diabetic ketoacidosis; HbA1c: Glycated hemoglobin; PPI: Proton pump inhibitor

Year	Age	Sex	DKA	pH	HbA1c (%)	Complaint	Treatment	Progress
2006	37	F				Vomiting, diarrhea	PPI	Healing
2007	33	M	+	7.315	11.3	Disturbance of consciousness, vomiting blood	PPI	Healing
2008	67	F	+	7.165	6.8	Disturbance of consciousness	PPI	Healing
2010	41	M	+	7.169	11.9	Physical weariness, diarrhea	PPI	Healing
2010	71	F	+	7.317	9.5	Physical weariness, vomiting blood	PPI, sucralfate hydrate	Healing
2014	38	M				Vomiting blood	PPI	Healing
2016	76	M	-	7.439		Vomiting blood	PPI	Healing
2023	49	M	+	7.317	7.3	Vomiting blood	PPI	Healing

Among the six cases with available blood data, all except one were complicated with DKA, suggesting that the aforementioned mechanisms may play a role in ANE development. The diagnosis was confirmed based on the clinical course of the disease, the presence of blackened esophageal mucosa on upper gastrointestinal endoscopy, and observation of necrotic esophageal mucosa on histopathological examination [1]. The cause of the blackening of the esophageal mucosa is thought to be either blood coagulation in the necrotic tissue or exposure to gastric acid [14]. In an autopsy case, characteristic dark-colored mucosa was observed in the ANE, and pathological examination revealed no malignant findings. However, there was evidence of inflammatory cell infiltration and granulation tissue with prominent hypervascularization, which supported ANE diagnosis. Lesions are typically found in the distal two-thirds of the esophagus; however, in our case, lesions were observed from the middle of the esophagus to the esophagogastric junction. This distribution is believed to result from reduced nutritional blood flow compared to the proximal one-third, which receives blood supply from the superior and inferior thyroid arteries [4]. The fact that esophageal inflammation is interrupted at the borders between the squamous and columnar epithelia suggests the involvement of gastric acid. Treatment typically involves fasting, central intravenous nutrition, and PPI use. ANE prognosis is influenced by the underlying disease. Gurvits et al. [15] reported a mortality rate of 31.8%, indicating a poor prognosis. However, deaths due to ANE are uncommon. The prognosis is generally good if the disease progresses without complications, and the time until meal resumption is typically approximately 10 days [5]. In an autopsy case of ANE, the implementation of fasting management with central venous nutrition and PPI led to improvement. Our patient resumed eating on the 13th day of the illness. Of the eight cases that we were able to retrieve, all patients were treated with PPI. Furthermore, no deaths were reported, and all patients recovered without complications, indicating a relatively favorable prognosis (Table 2). The most common complication of this syndrome is esophageal stricture, which occurs in 10-25% of patients. Typically, esophageal strictures develop within 7-10 days. However, there is a reported case in which the onset of esophageal stricture occurred after approximately one month. Therefore, careful follow-up of patients for an extended period is necessary to monitor delayed complications.

## Conclusions

We encountered a case of DKA complicated by necrotizing esophagitis. In DKA, gastric dilatation, atony, and rapid hyperglycemia can lead to a decrease in lower esophageal pressure and abnormal esophageal peristalsis. Additionally, hyperglycemia can result in delayed gastric emptying, even in the absence of autonomic nervous system disorders, causing gastroprokinetic disorders. The combination of these factors, along with increased intraesophageal pressure, reflux of gastric acid into the esophagus, dehydration-induced ischemia of the esophageal mucosa, and microcirculatory disturbances in the lower esophagus due to DKA, may contribute to the development of ANE. While rare, it is recommended to perform upper gastrointestinal endoscopy aggressively when DKA is associated with gastrointestinal symptoms and vomiting with coffee residue-like vomiting.
